# Characterization of the mitochondrial genome of *Megalobrama terminalis* in the Heilong River and a clearer phylogeny of the genus *Megalobrama*

**DOI:** 10.1038/s41598-019-44721-2

**Published:** 2019-06-11

**Authors:** Xuesong Hu, Peixian Luan, Chunhua Cao, Chitao Li, Zhiying Jia, Yanlong Ge, Mei Shang, Shihui Wang, Zining Meng, Jingou Tong, Lianyu Shi

**Affiliations:** 1 National and Local United Engineering Laboratory for Freshwater Fish Breeding, Heilongjiang River Fisheries Research Institute, Chinese Academy of Fishery Sciences, Harbin, 150070 China; 2 State Key Laboratory of Biocontrol, Institute of Aquatic Economic Animals and the Guangdong Province Key Laboratory for Aquatic Economi Animals, School of Life Sciences, Sun Yat-Sen University, Guangzhou, 510275 China; 3 State Key Laboratory of Freshwater Ecology and Biotechnology, Institute of Hydrobiology, Chinese Academy of Sciences, Wuhan, 430072 China

**Keywords:** Conservation biology, Genetic markers

## Abstract

*Megalobrama terminalis* distributed in Sino-Russian Heilong-Amur River basin has decreased dramatically in the last few decades. It has been listed in the Red Book of the Russian Federation as an endangered fish species. Here, the complete mitochondrial (mt) genome sequence of *M. terminalis* in the Heilong River (MTH) was first determined and characterized. Additionally, we identified a population-specific single nucleotide polymorphism (SNP) locus in MTH which could effectively separate MTH from the six other populations of the genus *Megalobrama* in the absence of hybridization. Moreover, phylogenetic analyses determined that the Xi River *M. hoffmanni* is located at the basal branch of the clade, and the rest of the group is divided into two assemblages, namely, one containing *M. terminalis* from Qiantang River and Jinsha River Reservoir/Longxi River *M. Pellegrini*/MTH and the other containing *M. amblycephala* from Liangzi Lake and Yi River. We clarify the intraspecies identity of MTH and construct a clearer phylogeny of the genus *Megalobrama*, which will contribute to the germplasm identification, protection and development of MTH in the future.

## Introduction

**Figure 1:**
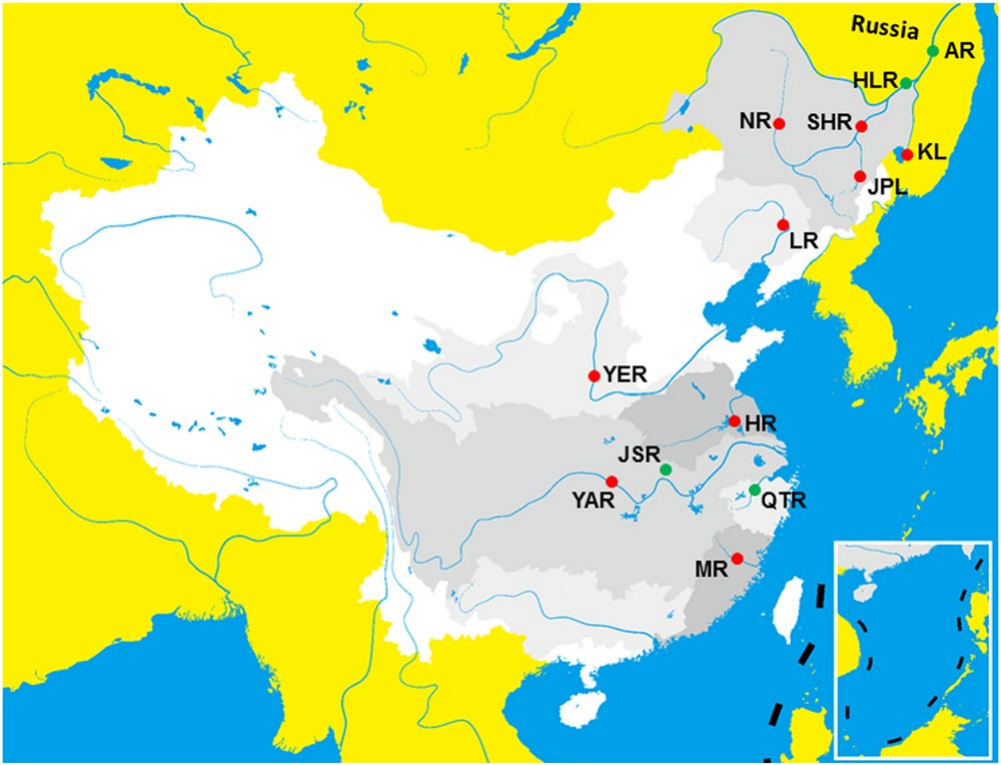
The historical distribution of *M. terminalis*. YAR, Yangtze River; MR, Min River; QTR, Qiantang River; JSR, Jinsha River Reservoir; HR, Huai River; YER, Yellow River; LR, Liao River; HLR, Heilong River (Fuyuan); AR, Amur River; SHR, Songhua River; NR, Nen River; JPL, Jingpo Lake; KL, Khanka Lake. A red dot represents that wild *M. terminalis* in the location has disappeared. A green dot indicates that wild *M. terminalis* in the location is still present.

The genus *Megalobrama* belongs to Cultrinae in Cyprinidae, and is one of the most economically important fish genera in China. This genus contains four main species: *M. amblycephala, M. pellegrini, M. hoffmanni and M. terminalis*^1,2^. The most widely distributed species among these is *M. terminalis*. Historically, *M. terminalis* inhabited the middle and upper reaches of the Yangtze, Heilong River Basin, Min, Qiantang, Huai, Yellow and Liao Rivers^1,3,4^ (Fig. 1). Notably, there is only one wild population in the Jinsha River Reservoir of Hongan County in Hubei Province^5^, but its origin is unclear.

To date, most of the wild populations of *M. terminalis* that lived in the original habitats have disappeared except for those in the Qiantang River, Heilong River and Jinsha River Reservoir (Fig. 1). The Heilong River is a Sino-Russian border river, and is known as the Amur River in Russia. *M. terminalis* in the Amur River has been listed as endangered in the Red Book of the Russian Federation^6^. In the Heilong River Basin, *M. terminalis* is called Faluo and lives in several water systems^7^, including the Heilong River, Songhua River, Nen River, Jingpo Lake, and Khanka Lake (Fig. 1)^3,4^. Faluo is a famous endemic fish species with several desirable qualities, such as a large body size, high intramuscular fat content and delicious taste. Since the 1960s, however, overfishing and pollution have caused the habitats of Faluo to decrease dramatically^7^. Currently, only dozens of individuals of Faluo can be captured per year near Fuyuan city (134°28′E, 48°37′N) (Fig. 1), which is located in Heilongjiang Province. Faluo is easy to catch due to its body shape and has late sexual maturity (more than five years)^4,6^. Therefore, its population growth is not fast enough to confer resilience, increasing the risk of complete extirpation resulting from environmental changes and anthropogenic factors.

As Faluo is the only fish species of the genus *Megalobrama* that lives in the northern Yellow River, some morphological characters of Faluo in this region are different from those of populations of *M. terminalis* in southern China. Accordingly, some researchers have questioned the intraspecific taxonomic relationship between Faluo and southern *M. terminalis*^7^. So far, few data have been reported on the genetics and genomics of Faluo because of the difficulty in sampling. Evidence reflecting the molecular variations in Faluo and the phylogenetic relationships between Faluo and other fish species within the genus *Megalobrama* is scarce. On the other hand, regarding the interspecific relationships within the genus *Megalobrama*, most of the available data suggest close relationships among *M. terminalis*, *M. pellegrini* and *M. amblycephala*, and *M. hoffmanni* is more distantly related to the other species^8–14^. However, the relationships between *M. terminalis*, *M. pellegrini* and *M. amblycephala* have previously been debated. In a phylogenetic study of Eastern Asian Cyprinidae based on mitochondrial CYTB, *M. terminalis* and *M. amblycephala* are sister taxa that form a clade with *M. pellegrini*^8^. In contrast, in another study based on mitochondrial CYTB, Xie *et al*. proposed that *M. terminalis* was closely related to *M. pellegrini* but not to *M. amblycephala*^9^. In a subsequent study based on mitochondrial COI and ND2, Bai *et al*.^10^ supported the proposal of Xie *et al*.^9^. To some extent, the divergence in conclusions about the interspecific relationships within the genus *Megalobrama* interferes with our understanding of the phylogenetic position of Faluo within the genus *Megalobrama*.

Recently, increasing evidence has shown that the whole mt genome could reveal more accurate phylogenetic relationships with higher resolution than single genes or segments^11,15–18^. Here, we obtained the first complete mitochondrial (mt) genome sequence of Faluo in the Heilong River (MTH) and confirmed that the sequence (GenBank: AB626850) of *M. terminalis* (in the Amur River of Russia, Fig. 1) submitted by Imoto *et al*.^19^ was from *Parabramis pekinensis strenosoma* which had similar morphological features with *M. terminalis*^10^. Furthermore, we also isolated the complete mt genomes of *M. terminalis* from the Qiantang River (MTQ) and Jinsha River Reservoir (MTJ), and of *M. amblycephala* from Liangzi Lake (MAL) and Yi River (MAY). The single nucleotide variations, specific markers and phylogeny between MTH and six other representative geographic samples within the genus *Megalobrama* were analyzed from a mt genome perspective.

## Materials and Methods

### Sample collection and DNA extraction

**Figure 2:**
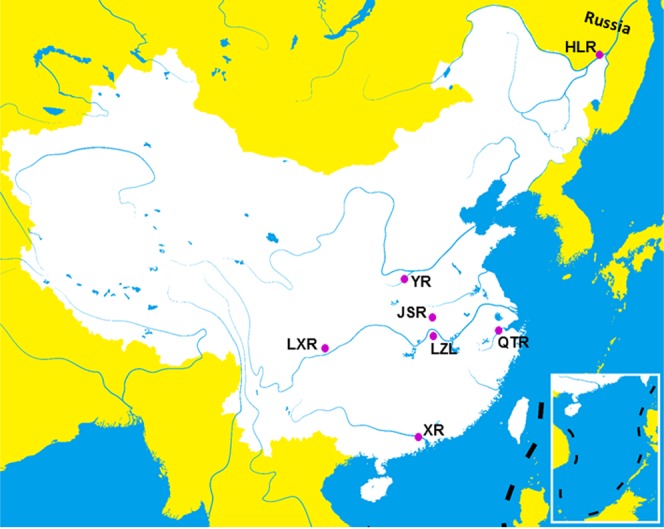
Sampling locations of fish in the genus *Megalobrama*. HLR, Heilong River; QTR, Qiantang River; JSR, Jinsha River Reservoir; YR, Yi River; LZL, Liangzi Lake; LXR, Longxi River; XR, Xi River.

A total of 210 fish from 7 populations (30 per population) of 4 species in the genus *Megalobrama* were sampled. Sampling locations are shown in Fig. 2. Total DNA was extracted from fin tissues from all samples for each population using the phenol/chloroform method^20^. All animal experiments were approved and conducted in accordance with the guidelines of the Animal Research and Ethics Committees of Heilongjiang River Fisheries Research Institute.

### Primer design, PCR amplification and sequencing

The complete mt genomic sequences from two species in the genus *Megalobrama*, namely, *M. pellegrini* from the Longxi River (MPL) and *M. hoffmanni* from the Xi River (MHX), were determined in a previous study^11^. Thus, one genomic sample from each population (MTH, MTQ, MTJ, MAL and MAY) was used as the template for whole mt genome sequencing. A pair of primers (mt F1 and mt R1) described by Huang *et al*.^21^ was used to amplify the D-loop region. Twenty-one other pairs of primers (Table 1) with putative overlapping amplification regions were designed using Primer 5.0 (Premier Biosoft International, Palo Alto, CA, USA) according to the complete mt genome sequences of species in the genus *Megalobrama* available in GenBank. The PCR conditions were as follows: 94 °C for 5 min, followed by 30 cycles at 94 °C 30 s, 59 °C for 15 s, and 72 °C for 45 s, and an extension at 72 °C for 5 min. PCR components contained 13.7 µL H_2_O, 2 µL 10 × PCR buffer, 1.6 µL 1.5 mM MgCl_2_, 0.6 µL of each primer (10 µM), 0.4 µL 10 mM dNTPs, 0.1 µL *Taq* DNA polymerase (5 U/µL) and 1 µL DNA template (50 ng/µL) in a total volume of 20 µL. PCR products were separated on 1.0% of agarose gels with 0.5 µg/mL of ethidium bromide. The DNA fragments were purified using an E. Z. N. A. Gel Extraction Kit (Omega Bio-Tek, Norcross, GA, USA) and subcloned into pMD18-T vectors (TaKaRa, Shiga, Japan). Recombinant clones containing each target fragment were sequenced on an ABI 3700 sequencer (Applied Biosystems, PerkinElmer, Foster City, CA, USA).

**Table 1:** The twenty-two pairs of primers used to amplify the complete mitochondrial genome of *Megalobrama terminalis* and *M. amblycephala*. Mixed bases: R: A/G.

Primers	Sequences (from 5′ to 3′)	Primers	Sequences (from 5′ to 3′)
mt F1	CACCCCTGGCTCCCAAAGCCA	mt R1	RCTGCGGAGACTTGCATGTGTAA
mt F2	GCGTAGCTTAATGCAAAGCATAAC	mt R2	GCCCATTTCTTCCCACTTCGT
mt F3	CTATATACCGCCGTCGTCAGC	mt R3	CTATCCATTTCTCAGGCAACCAG
mtF4	CTAACCCGTCTCTGTGGCAAAAG	mt R4	TAAGCCCTCGTTTAGCCATTCAT
mt F5	TGCCCAGTGACTACAGGTTC	mt R5	TGAACTGGGAAGAGGATTTG
mtF6	CGCCCGAAATAAGGACATACT	mt R6	TCGTATCGGAATCGTGGGTA
mt F7	CACCACATCCCAGAATTAACAAC	mt R7	GGTTTGGTTTAGACCTCCTCATC
mt F8	CCCCCTTCGCCCTAATTATTC	mt R8	ATGGATGCTCGCTGGCTTGA
mt F9	GTCCCTGATAAGACCTACAAGAG	mt R9	GGGTCAAAGAATGTGGTGTT
mt F10	CGTACTTCTTCTCCTCTCCCTA	mt R10	GTCAGAGTATCGTCGTGGCA
mt F11	TTCACTGATTCCCCCTATTTAC	mt R11	CTACTATTCGGTGGTCTGTTTCT
mt F12	CCATGGGACATCAATGATACTG	mt R12	GGATGGGTGTTCCTTCTGGT
mt F13	GGCTTGCCACCGTGATTATTG	mt R13	TCTGCGATTGTGAAGGGTGCT
mt F14	AATCTCTTGCACTCACAATCCT	mt R14	AACTCCCTATTCTGCTCATTCTA
mt F15	CTGCCATGAGGAGACCAACT	mt R15	CAGGGTGTAGAATAGGAAATAGG
mt F16	GCTACACTCATCCCAACTCTTATC	mt R16	ATTAGTGTCCCTGCTCCTGTG
mt F17	CCATTAACAGCGGTATGATGATTC	mt R17	ACCTTCTCAGCCGATGAATAGC
mt F18	CACTCTGACCCAAACATGAAC	mt R18	AGACTGCGGTGAATGATGTG
mt F19	TTGGCAGCCTTGCATTAACAG	mt R19	TCTATGTTGCGTGGCGATGTT
mt F20	CACCCACGCACAAATAACCAAC	mt R20	GCGGTTGAAATGTCGGAGGT
mt F21	GTTGACCTTCCCACACCATCCA	mt R21	GGGCGGAATGTTAGTCCTCGTT
mt F22	CGATCCATCCCAAACAAACT	mt R22	GAGGGTGGAATCTTACAGTTATG

### Mitochondrial genome assembly and sequence comparison

Sequencing data and the boundaries from each segment with overlapping domains were checked to ensure authenticity. The assembled sequences were annotated by comparisons with known complete mitochondrial genomes of closely related species, including MPL and MHX. The secondary structures of tRNAs were identified using the web-based tRNA-scan SE 2.0 program (http://lowelab.ucsc.edu/tRNAscan-SE/)^22^. Complete mt sequences from MTH, MTQ, MTJ, MAL and MAY have been deposited in GenBank with the accession numbers listed in Table 2. Furthermore, complete mt genome sequences from *M. terminalis* in the Amur River in Russia (MTA)^19^ and *Parabramis pekinensis strenosoma* in the Heilong River (PPH)^23^ were used in the sequence correction (Table 2). The mt genome comparisons between MTH and MTQ, MTJ, MPL, MAL, MAY, MHX, MTA and PPH were performed by BLAST using the CGView Comparison Tool (CCT)^24^.

**Table 2:** Information on sampling and mitochondrial genome sequences used in this study.

Species	Code	Origin	Size (bp)	Accession no.	Reference
**Ingroup**					
*Megalobrama terminalis*	MTH	Heilong River, China	16,621	MH289765	This study
	MTQ	Qiantang River, China	16,621	MH289767	This study
	MTJ	Jinsha River Reservoir, China	16,621	MH289766	This study
*Megalobrama* *pellegrini*	MPL	Longxi River, China	16,621	JX242529	Lai *et al*.^11^
*Megalobrama amblycephala*	MAL	Liangzi Lake, China	16,623	MH289764	This study
	MAY	Yi River, China	16,623	MH289763	This study
*Megalobrama hoffmanni*	MHX	Xi River, China	16,622	JX242530	Lai *et al*.^11^
**Sequences for genome correction**					
*Megalobrama* *terminalis*	MTA	Amur River, Russia	16,623	AB626850	Imoto *et al*.^19^
*Parabramis pekinensis strenosoma*	PPH	Heilong River, China	16,623	KF857485	Duan *et al*.^23^
**Outgroup**					
*Culter dabryi*		—	16,622	NC_021418	Zhang *et al*.^25^
*Culter mongolicus*		—	16,622	AP009060	Saitoh *et al*.^18^

### Specific SNP loci identification

Single nucleotide polymorphism (SNP) loci of the complete mt genomes between MTH and other species from the genus *Megalobrama* were identified based on comparisons performed with the Clustal W method using the MegAlign program in DNASTAR Lasergene 7.1 software (DNASTAR Inc., Madison, WI, USA). Moreover, a pair of primers (forward: 5′-GCATCTGGCTTCA-ATCTCA-3′; reverse: 5′-GCGTAGGGGATTTGCTGA-3′) was designed to amplify a putative fragment of 380 bp containing the specific locus SNP locus C549T in the D-loop region in MTH based on the 349–728 bp domain of the MTH mt genome in GenBank (Accession No: MH289765). The PCR components were the same as those mentioned above, and the conditions of the reaction were as follows: 94 °C for 5 min, followed by 30 cycles at 94 °C 30 sec, 56 °C for 30 sec, and 72 °C for 30 sec, and an extension at 72 °C for 5 min. After PCR, 2 µL of RE × 10 buffer, 0.2 µL acetylated BSA and 5 U *Taq*I (Promega Co., USA) was added to each sample (~1 µg), and the reaction was incubated at 65 °C for 2 h. Then, all digested samples of 7 populations (30 per population) were separated on 4% agarose gels with 0.5 µg/mL ethidium bromide.

### Phylogenetic analysis

All 7 complete mt genomes from the ingroup (MTH, MTQ, MTJ, MPL, MAL, MAY and MHX) and the outgroup consisting of genus *Culter* (*Culter dabryi*^25^ *a*nd *Culter mongolicus*^18^) (Table 2) were aligned using the Clustal W algorithm^26^ implemented in the MEGA 7 program^27^. Substitution saturation was evaluated by DAMBE 6.4.1^28^. Phylogenetic analyses were conducted based on complete nucleotide sequences using maximum likelihood (ML), maximum parsimony (MP) and Bayesian inference (BI) methods. ML and MP calculations were completed using MEGA 7 with 1000 bootstraps^27^. The best model (HKY + G) for the ML analysis was estimated based on the Bayesian information criterion (BIC) in this program. Bayesian phylogenetic analysis was performed using MrBayes 3.2^29^. The best model (GTR + I) was selected based on Akaike information criterion (AIC) in the Modeltest 3.7 program^30^. Two million generations with four chains were run, sampling per 500 generations and discarding the initial 25% of samples as a burn-in.

## Results

### Characteristics of the MTH mt genome

**Figure 3:**
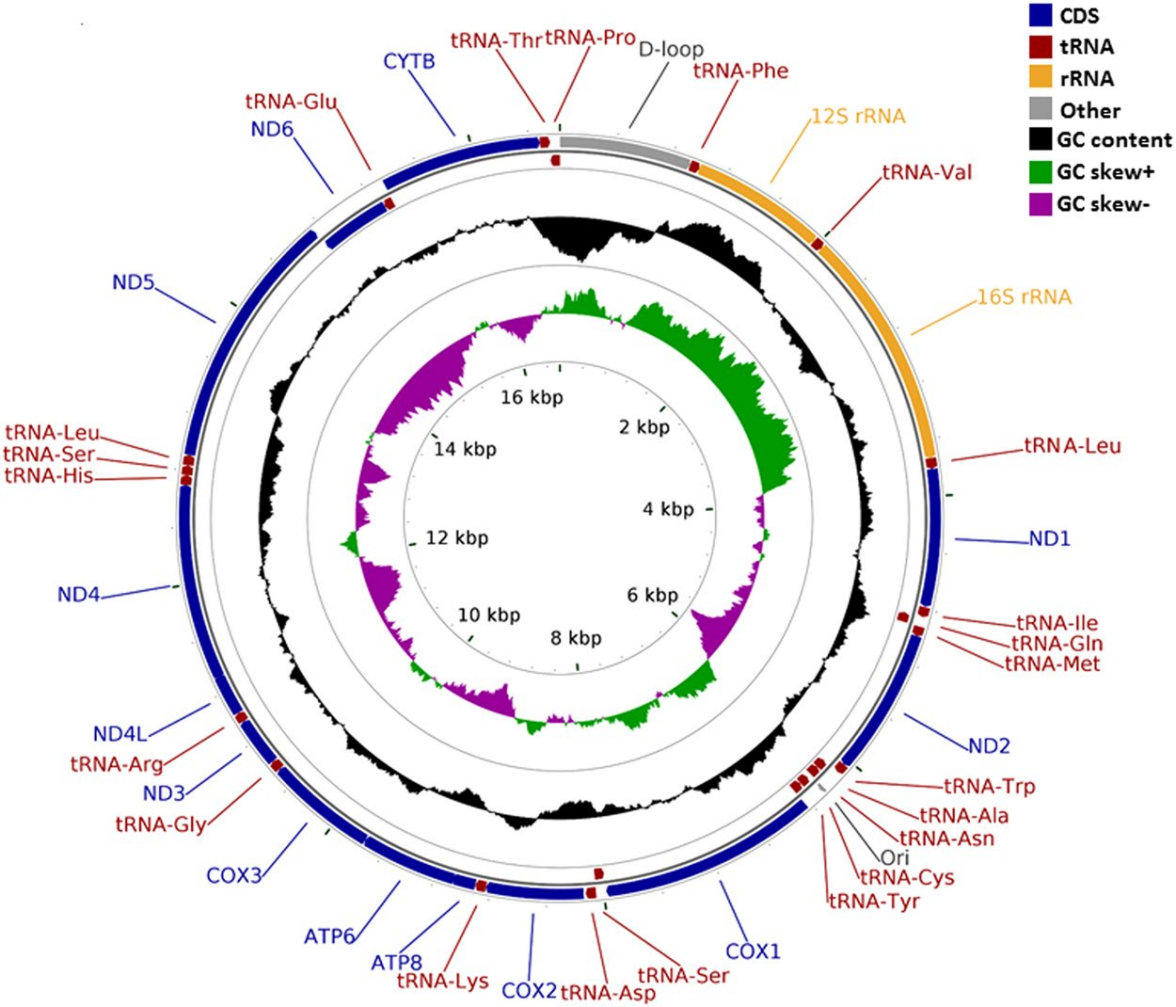
Circular map of the mitochondrial genome of *M. terminalis* in the Heilong River (MTH). Genes encoded by heavy and light strands with inverse arrow directions are shown outside and inside the circle, respectively. The black ring indicates the GC content (outward and inward peaks showing above or below average GC content, respectively). The purple-green ring indicates the GC skew [(G − C)/(G + C), purple (between 0 and 1), green (between −1 and 0)].

We determined the complete mt genome of MTH. The assembled mt genome of MTH had a typical circular structure with a length of 16,621 bp, which is similar (16,621–16,623 bp) to that of its closely related species in the genus *Megalobrama* (Table 3). The whole genome contains 13 protein-coding genes, 22 tRNA genes, 2 rRNA (12SrRNA and 16SrRNA), and a putative control region (Table 3). The heavy (H) strand encodes 28 genes, while the light (L) strand encodes 9 genes (Fig. 3, Table 3). Based on prediction by tRNAscan-SE, 21 of 22 tRNAs fold into the typical cloverleaf secondary structure, while tRNA^Ser(AGN)^ lacks the dihydrouridine (DHU) stem. ATG is the major start codon of the protein-coding genes, and only one start codon (GTG) is used in the COXI gene. In 13 protein-coding genes, seven (ND1, COX1, ATP8, ATP6, ND4L, ND5, and ND6) have a TAA stop codon, while the other six have incomplete stop codons of TA (COX3 and ND4) and T (ND2, COX2, ND3, and CYTB) (Table 3). The overall GC content of the MTH mt genome is 44.1%, while that of the D-loop region is only 35.9% (Fig. 3 and Table 4).

**Table 3:** Location and arrangement of genes in the 16,621 bp mitochondrial genome of *Megalobrama terminalis* in the Heilong River (MTH).

Gene	Strand	Gene					Intergenic spacer
		From (bp)	To (bp)	Size (bp)	Start codon	Stop codon	
*D-loop*	—	1	937	937			
*tRNA* ^*Phe*^	H	938	1006	69			
*12SrRNA*	H	1007	1967	961			
*tRNAVal*	H	1968	2039	72			
*16SrRNA*	H	2040	3730	1691			1
*tRNALeu*	H	3732	3807	76			1
*ND1*	H	3809	4783	975	ATG	TAA	4
*tRNAIle*	H	4788	4859	72			−2
*tRNAGln*	L	4858	4928	71			1
*tRNAMet*	H	4930	4998	69			
*ND2*	H	4999	6043	1045	ATG	T-	
*tRNATrp*	H	6044	6114	71			1
*tRNAAla*	L	6116	6184	69			1
*tRNAAsn*	L	6186	6258	73			
*OL*	—	6259	6290	32			
*tRNACys*	L	6291	6358	68			2
*tRNATyr*	L	6361	6431	71			1
*COX1*	H	6433	7983	1551	GTG	TAA	
*tRNASer*	L	7984	8054	71			2
*tRNAAsp*	H	8057	8130	74			13
*COX2*	H	8144	8834	691	ATG	T-	
*tRNALys*	H	8835	8910	76			1
*ATP8*	H	8912	9076	165	ATG	TAA	−7
*ATP6*	H	9070	9753	684	ATG	TAA	
*COX3*	H	9753	10,537	785	ATG	TA-	
*tRNAGly*	H	10,538	10,609	72			
*ND3*	H	10,610	10,958	349	ATG	T-	
*tRNAArg*	H	10,959	11,028	70			
*ND4L*	H	11,029	11,325	297	ATG	TAA	−7
*ND4*	H	11,319	12,700	1382	ATG	TA-DNACO	
*tRNA* ^*His*^	H	12,701	12,769	69			
*tRNA* ^*Ser*^	H	12,770	12,838	69			1
*tRNA* ^*Leu*^	H	12,840	12,912	73			
*ND5*	H	12,913	14,748	1836	ATG	TAA	−4
*ND6*	L	14,745	15,266	522	ATG	TAA	
*tRNA* ^*Glu*^	L	15,267	15,335	69			4
*CYTB*	H	15,340	16,480	1141	ATG	T-	
*tRNA* ^*Thr*^	H	16,481	16,552	72			
*tRNA* ^*Pro*^	L	16,552	16,621	70

**Table 4:** Base composition (%) in the mitochondrial genome of *Megalobrama terminalis* in the Heilong River (MTH).

	A	T	G	C
*D-loop*	33.4	30.7	14.2	21.7
*12sRNA*	30.6	18.9	23.0	27.5
*16sRNA*	36.7	20.2	20.3	22.8
*ND1*	29.9	24.9	15.3	29.8
*ND2*	32.1	23.2	12.9	31.9
*COX1*	27.5	28.4	17.4	26.8
*COX2*	30.0	26.0	16.5	27.5
*ATP8*	29.4	21.8	9.1	29.7
*ATP6*	28.7	28.2	13.6	29.5
*COX3*	27.8	26.1	17.3	28.8
*ND3*	27.5	25.2	14.6	32.7
*ND4L*	26.3	25.9	15.8	32.0
*ND4*	31.4	26.2	13.6	28.8
*ND5*	31.6	24.8	12.8	30.8
*ND6*	42.5	13.0	11.9	32.6
*CYTB*	29.0	27.4	14.9	28.7
Total	31.2	24.8	16.2	27.9

### Comparison with closely related species of Megalobrama and Parabramis

**Figure 4:**
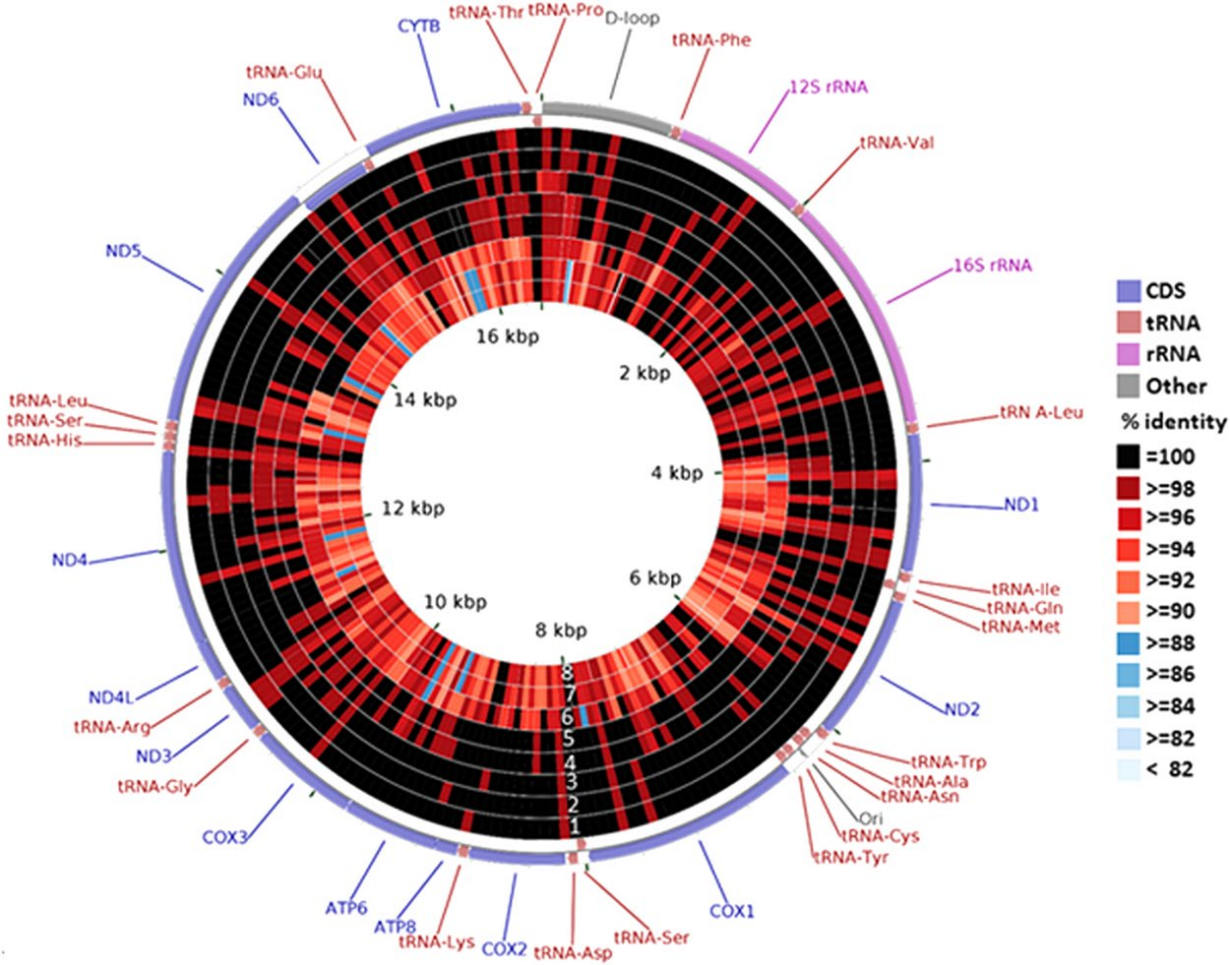
Graphical map of the nucleotide percent identity of mitochondrial genomes determined by BLAST between *Megalobrama terminalis* in the Heilong River (MTH) and other fish of the genus *Megalobrama* and between MTH and the closely related *Parabramis pekinensis strenosoma*. The rings labeled 1 to 8 represent the BLAST results of sequences from MTH against those from *M. terminalis* in the Qiantang River (MTQ), *M. terminalis* in the Jinsha River Reservoir (MTJ), *M. pellegrini* in the Longxi River (MPL), *M. amblycephala* in the Yi River (MAY), *M. amblycephala* in the Liangzi Lake (MAL), *M. hoffmanni* in the Xi River (MHX), *P. pekinensis strenosoma* in the Heilong River (PPH) and *M. terminalis* in the Amur River (MTA).

We compared the percent identity and pairwise distances of mitogenomes among MTH, MTQ, MTJ, MAL, MAY, MTA and PPH. The number of the percent identity results were showed in Table 5, and the sequences identity comparison results were reflected in the CCT BLAST map (Fig. 4). High identity was found between MTH and MTQ, MTJ, and MPL (99.6–99.7%). The identity was low between MTH and MHX (96.1%), and the lowest identities were observed between MTH and MTA and between MTH and PPH (95.2% each). However, MTA had high identity with PPH (99.7%). Seventeen specific SNPs were detected between MTH and other fish species in the genus *Megalobrama*. The numbers of specific SNPs of MTQ and MTJ were 8 and 11, respectively (Table 6). Thirteen MTH-specific SNPs were found in coding regions (ND1, 2; ND2, 1; Cox1, 1; CoxII, 1; CoxIII, 1; ND3, 1; ND4, 2; ND5, 3; and ND6, 1). Only three nonsynonymous SNPs are found, and they were located in ND1 (nucleotide location 4,542 in the MTH assembly and with a codon change from GTC^Val^ to GCC^Ala^), COXIII (nucleotide location 10,180 in the MTH assembly and with a codon change from AAG^Lys^ to ACG^Thr^) and ND4 (nucleotide location 11,400 in the MTH assembly and with a codon change from GCC^Ala^ to ACC^Thr^) (Table 7). There were four MTH-specific SNPs in noncoding regions (D-loop, 1; 12SrRNA, 1; and 16SrRNA, 2) (Table 7).

**Table 5:** Percent identities based on complete mitochondrial genomes among MTH, MTQ, MTJ, MAL, MAY, MPL, MHX, MTA and PPH.

Population	MTH	MTQ	MTJ	MPL	MAL	MAY	MHX	MTA	PPH
MTH	—	99.7	99.7	99.6	99.2	99.1	96.1	95.2	95.2
MTQ		—	99.8	99.7	99.2	99.1	96.1	95.2	95.2
MTJ			—	99.7	99.1	99.1	96.1	95.2	95.2
MPL				—	99.0	99.0	96.0	95.2	95.1
MAL					—	99.9	96.0	95.2	95.1
MAY						—	96.0	95.2	95.2
MHX							—	94.9	94.9
MTA								—	99.7
PPH									—

GenBank accession numbers: MTH, *Megalobrama terminalis* in the Heilong River (MH289765); MTQ, *M. terminalis* in the Qiantang Rive (MH289767); MTJ, *M. terminalis* in the Jinsha River Reservoir (MH289766); MPL, *M. pellegrini* in the Longxi River (JX242529); MAL, *M. amblycephala* in the Liangzi Lake (MH289764); MAY, *M. amblycephala* in the Yi River (MH289763); MHX, *M. hoffmanni* in the Xi River (JX242530); MTA, *M. terminalis* in the Amur River (AB626850); PPH, *Parabramis pekinensis strenosoma* in the Heilongjiang River (KF857485).

**Table 6:** The number of specific SNPs in the mitochondrial genomes of MTH, MTQ, MTJ, MPL MAL, MAY and MHX. MTH, *Megalobrama terminalis* in the Heilong River; MTQ, *M. terminalis* in the Qiantang Rive; MTJ, *M. terminalis* in the Jinsha River Reservoir; MPL, *M. pellegrini* in the Longxi River; MAL, *M. amblycephala* in the Liangzi Lake; MAY, *M. amblycephala* in the Yi River; MHX, *M. hoffmanni* in the Xi River.

Gene	MTH	MTQ	MTJ	MPL	MAL	MAY	MHX
*D-loop*	1	0	1	3	0	0	29
*12sRNA*	1	0	0	2	0	0	24
*16sRNA*	2	0	0	1	0	0	25
*ND1*	2	2	1	1	0	0	53
*ND2*	1	1	1	2	0	0	54
*COX1*	1	0	1	1	1	0	43
*COX2*	1	0	0	1	1	0	26
*ATP8*	0	0	0	0	0	0	5
*ATP6*	0	0	1	0	0	1	39
*COX3*	1	0	0	1	0	0	32
*ND3*	1	1	0	2	0	0	16
*ND4L*	0	0	0	0	0	0	14
*ND4*	2	0	1	2	0	0	64
*ND5*	3	2	2	0	0	0	51
*ND6*	1	0	0	1	0	0	21
*CYTB*	0	2	3	2	0	0	69
Total	17	8	11	19	2	1	565

**Table 7:** Specific mitochondrial genome SNPs between *Megalobrama terminalis* in the Heilong River (MTH) and other *Megalobrama* fish (*M. terminalis* in the Qiantang River and Jinsha River Reservoir, *M. pellegrini* in the Longxi River, *M. amblycephala* in the Liangzi Lake and Yi River and *M. hoffmanni* in the Xi River).

Location in MTH^a^	Variation in MTH/others^b^	Amino acid variation in MTH/others^b^	Gene
549	C/T	—	*D-loop*
1718	A/G	—	*12SrRNA*
2673	T/A	—	*16SrRNA*
3387	A/G	—	*16SrRNA*
4061	CTA/TTA	L	*ND1*
4542	GTC/GCC	V/A	*ND1*
5394	ACC/ACA	T	*ND2*
7668	ATT/ATC	I	*COX1*
8158	ACA/ACG	T	*COX2*
10,180	AAG/ACG	K/T	*COX3*
10,831	CTT/CTC	L	*ND3*
11,400	GCC/ACC	A/T	*ND4*
12,698	TAT/TAC	Y	*ND4*
13,077	CCC/CCA	P	*ND5*
14,079	ACT/ACC	T	*ND5*
14,703	ATT/ATC	I	*ND5*
14,946	GGA/GGC	G	*ND6*

^a^Locations of polymorphic sites are in reference to the MTH assembly. ^b^Oblique lines (/) separate the variants of mononucleotides, codons, and amino acids in the involved genes between individuals from MTH and fish from other *Megalobrama* populations. Amino acids are indicated by capital letters corresponding to their one-letter code.

### Population-specific marker identification

**Figure 5:**
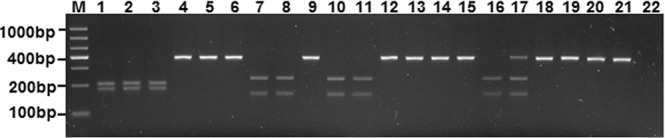
Differentiation between *Megalobrama terminalis* in the Heilongjiang (MTH) and other populations of the genus *Megalobrama* determined using polymerase chain reaction*-*restriction fragment length polymorphism of a D-loop region fragment with *Taq*I digestion. M = DL 1,000 DNA ladder, 1–3 = MTH, 4–6 = *M. pellegrini* in the Longxi River (MPL), 7–9 = *M. amblycephala* in the Liangzi Lake (MAL), 10–12 = *M. amblycephala* in the Yi River (MAY), 13–15 = *M. terminalis* in the Qiantang River (MTQ), 16–18 = *M. terminalis* in the Jinsha River Reservoir (MTJ), 19–21 = *M. hoffmanni* in the Xi River (MHX), 22 = Negative control.

The SNP C549T in the D-loop region formed one *Taq*I restriction site (T/CGA or TTGA) in the MTH population with one genotype (180/200 bp, T/CGA) (Fig. 5). In the six other populations, the base at the 549 site was T. However, only in the populations MTQ, MPL and MHX, there was one genotype (380 bp, TTGA) (Fig. 5). In the populations MTJ, MAL and MAY, there was one *Taq*I restriction site (T/CGA or TCAG) resulting from SNP G573A and SNP A574G, which is different from the position in MTH. In the MTJ population, there were three genotypes (157/223 bp; 157/223/380 bp; 380 bp). In populations MAL and MAY, there were two genotypes (157/223 bp or 380 bp) (Fig. 5).

### Phylogenetic analysis

**Figure 6:**
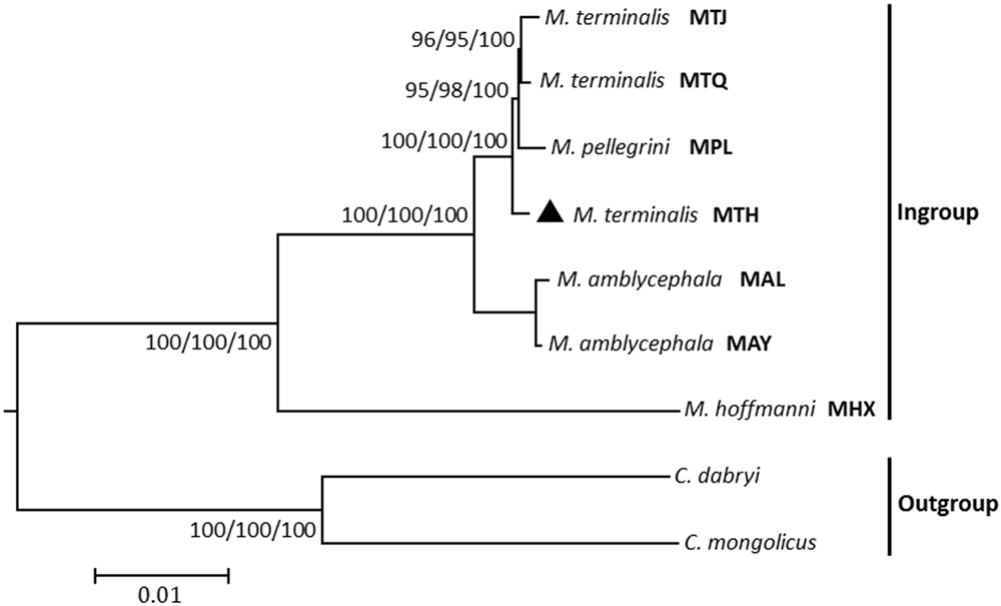
Phylogenetic tree of the genus *Megalobrama* (ingroup) inferred by using the maximum likelihood (ML) method based on the complete mitochondrial genome data. Values shown at each node of the tree correspond to the ML bootstrap values, maximum parsimony bootstrap values and Bayesian posterior probabilities given in percentages. *Culter dabryi* and *Culter mongolicus* were used as the outgroup. GenBank accession numbers: MTH, *Megalobrama terminalis* in the Heilong River (MH289765); MTQ, *M. terminalis* in the Qiantang Rive (MH289767); MTJ, *M. terminalis* in the Jinsha River Reservoir (MH289766); MPL, *M. pellegrini* in the Longxi River (JX242529); MAL, *M. amblycephala* in the Liangzi Lake (MH289764); MAY, *M. amblycephala* in the Yi River (MH289763); MHX, *M. hoffmanni* in the Xi River (JX242530); C. dabryi (NC_021418); C. mongolicus (AP009060).

The substitution saturation of all nucleotide sequences was not tested. The maximum parsimony, maximum likelihood, and Bayesian methods produced identical tree topologies with strong bootstrap and posterior probability values, and the maximum likelihood tree is shown here (Fig. 6). *M. terminalis* (MTH, MTQ and MTJ), *M. pellegrini* (MPL), *M. amblycephala* (MAL and MAY) and *M. hoffmanni* (MHX) composed a monophyletic clade. MHX was located at the basal branch of this clade. MTH, MTQ, MTJ and MPL formed one subclade, and MAL and MAY formed the other subclade. MTA, MTQ and MPL constituted a monophyletic clade. MTH has a sister relationship with a clade comprising MTQ, MTJ, and MPL.

## Discussion

Imota *et al*. submitted the full mtDNA sequence of *M. terminalis* in the Amur River (MTA) in Russia^19^. Based on a comparative analysis, however, we found that the lengths of the complete mt genomes of MTA, MHX, and PPH were identical (16,623 bp), and this sequence of MTA showed higher identity with that of PPH (99.7%) than with that of MTH or other *M. terminalis* (95.2%). In contrast, the length of the mt genome sequence (MTH) obtained in the present study was identical to those of MTQ, MTJ and MPL (16,621 bp), and showed a high identity with them (99.6–99.8%). These results suggest that the *M. terminalis* sequence provided by Imoto *et al*. was from PPH. *M. terminalis* and PPH have the same distribution in the Heilongjiang-Amur River basin and share some morphological and biological characteristics^31,32^. Thus, the confusion between the two is very easy to happen.

Furthermore, to understand the characteristics of the mt genome of MTH, we detected 17 SNP sites of specific to MTH, which was more than the number obtained for conspecific MTQ (8) and MTJ (11). In China, all fish species in the genus *Megalobrama* inhabit the southern Yellow River except Faluo, which lives in the Heilongjiang basin^1,32^, where the effective growing season of fish is only ~4 months, and the overwintering period beneath ice reaches ~5 months^33^. Therefore, unique habitats and long-term geographical isolation may have led to MTH having more polymorphic sites in its mt DNA than intraspecific MTQ and MTJ. Three nonsynonymous SNPs in ND1, COX3 and ND4 discovered between MTH and other fish species in the genus *Megalobrama* remain to be elucidated. Based on the comparative data of specific SNPs, we identified one population-specific marker in the D-loop region (C 549 T), which effectively separated MTH from the six other fish species in the genus *Megalobrama* in the absence of hybridization. All individuals of MTH used in the present study were homozygous at the specific marker, suggesting that MTH still has a relatively pure gene pool. The genus *Megalobrama* emerged relatively recently in Cyprinidae fish species^8,34^. *M. terminalis*, *M. amblycephala* and *M. pellegrini* are very similar in morphology before sexual maturity, and can hybridize with each other by artificial fertilization^35–37^. Recently, a culture variety of *M. amblycephala* bred by Li *et al*. began to appear in the aquaculture waters of northeast China^38^, and posed a potential threat to precious *M. terminalis* resources. Thus, further work is required to develop population-specific markers at the nuclear gene level for germplasm purity identification of MTH during sample collection and artificial breeding.

The species taxonomy of Faluo has yet to be fully accepted^7^. One of the major reasons is that Faluo shows some distinct morphological characters (number of vertebrae, length of the intestine/body length, body length/eye diameter, and hypopharyngeal teeth type) from southern *M. terminalis*^7^. Additionally, Faluo was not used as a representative specimen of *M. terminalis* in the early classification of the genus *Megalobrama*^1^. In our present phylogenetic analysis, however, MTH, MTJ and MTQ were in a well-supported monophyletic group. Considering that MTH showed the highest percent identity with MTJ and MTQ, we speculate that the present species classification of Faluo is undisputable.

To the best of our knowledge, this is the first phylogenetic analysis including samples from three different geographical populations of *M. terminalis* with clear origins. MTH in particular represents a population distribution in northernmost China. In previous studies, however, samples were only taken from one population of *M. terminalis* other than MTH^8–14^. Notably, MTQ, MTJ and MPL constituted a monophyletic clade, shared a common ancestor with MTH, and further clustered with MAL and MAY in the present study. This result clearly indicates that MPL rather than MTH has a close phylogenetic relationship with MTJ and MTQ. The present results give strong evidence by utilizing complete mt genome data, supporting *M. pellegrini* as one of the populations of *M. terminalis*.

The sequence of a complete mt genome from MTH was obtained for the first time. We expect the analysis of specific markers of MTH and identification at the population level will contribute to germplasm purity detection in future sampling and breeding practices. Furthermore, we confirmed that MTH belongs to the species *M. terminalis* by sequence comparisons and phylogenetic tree reconstruction. The introduction of MTH provided clearer phylogenetic relationships among species in the genus *Megalobrama* at the mitochondrial genome level, which will also be helpful in the protection and development of this endangered species in the future.

## Data Availability

The mitochondrial genome data of *Megalobrama terminalis* in the Heilong River (MH289765), Qiantang River (MH289767) and Jinsha River Reservoir (MH289766), and *Megalobrama amblycephala* in the Liangzi Lake (MH289764) and Yi River (MH289763) have been submitted to GenBank. The authors confirm that the data supporting the findings of this study are available within the article. However, any datasets generated and/or analysed during the current study are available from the corresponding author on reasonable requests.
